# Understanding evidence-informed decision-making: a rural interorganizational breastfeeding network

**DOI:** 10.1186/s12913-019-4138-6

**Published:** 2019-05-27

**Authors:** Sionnach Lukeman, Barbara Davies, Charmaine McPherson, Josephine Etowa

**Affiliations:** 1 St. Francis Xavier University, PO BOX 5000, Antigonish, Nova Scotia B2G 2W5 Canada; 2 Faculty of Health Sciences, University of Ottawa, 451 Smyth Road, Ottawa, Ontario, K1N 6N5 Canada; 3 Halifax, Nova Scotia, Canada

**Keywords:** Evidence-informed decision-making, Community networks, Case study, Leadership, Rural, Breastfeeding, Social network analysis, Knowledge mobilization

## Abstract

**Background:**

Networks are a vehicle for mobilizing knowledge, but there is little research about evidence-informed decision-making in community settings. Breastfeeding is a powerful intervention for population health; combined system and community interventions can increase exclusive breastfeeding rates by 2.5 times. This study examined evidence-informed decision-making within an interorganizational network, including the facilitators and barriers to achieving network goals.

**Methods:**

A mixed method case study design was used. The primary sources of data were focus group discussion and questionnaire administration. Data were analyzed concurrently using framework analysis and social network analysis.

**Results:**

Key findings were at the interorganizational and external levels: 1) Relationships and trust are connected to knowledge exchange 2) Need for multiple levels of leadership.

**Conclusions:**

The findings of this study have potential implications for enhancing the use of evidence-informed decision-making as other networks work toward Baby Friendly Initiative (BFI) designation and also highlights the potential for network maps to be used as a knowledge mobilization tool.

## Background

Existing approaches, frameworks and tools to understand evidence-informed decision-making (EIDM) in healthcare need further development within community-based settings [1, 2]. Best and colleagues outlined the link between EIDM and networks: “… we see knowledge products as embedded within relationships of linkage and exchange, which in turn are embedded within a larger system that is shaped by culture, priorities, and capacities (p. 628) [3]”. EIDM is the deliberate consideration and use of the best available evidence, including research, local data, and practice-related knowledge, when making decisions regarding practice, policy or programming [4, 5]. Networks are a potential vehicle for mobilizing knowledge, but there is little current research about facilitators and barriers in the community setting [6].

Interorganizational networks are an appropriate forum for addressing complex inter-related problems [7], and are defined as collaborative multi-disciplinary partnerships that represent different organizations, with members who are striving to achieve a common goal [8, 9]. Strong partnerships in rural areas enhance the participation of community members, which is important when designing interventions or approaching complex issues that require adaptation to the local context [10].

### An exemplar- breastfeeding promotion

Breastfeeding is an important intervention for the health of a population; promotion starts with the dissemination and use of robust evidence. Combined health system and community interventions, including Baby Friendly Initiative (BFI) designation, can increase exclusive breastfeeding rates by 2.5 times [11]. Human milk provides the optimal combination of nutritional, immunological, and emotional benefits, and it is recommended that healthy term infants be breastfed exclusively for the first 6 months of life and and to continue breastfeeding for up to 2 years [12]. There is a significant gap between these recommendations and the current rates in Canada, especially as you move from West to East [13, 14]. Overall, 89% of Canadian women in the years 2011–2012 initiated breastfeeding, but only 26% breastfed exclusively for 6 months [14]. In Nova Scotia between 2006 and 2009, 76% of women are initiating breastfeeding yet the exclusive breastfeeding duration rate for 6 months is only 11.4% [13].

WHO and UNICEF jointly developed a framework and accreditation systems to assist healthcare providers in promoting and supporting breastfeeding [15]. In addition to a ten-step process for hospitals and community organizations, the program recommends a multi-level, multi-sector effort coupled with legislative protection and social promotion to support optimal infant and child feeding practices [15]. A systematic review investigating the impact of BFI on breastfeeding and child outcomes suggests that not only does BFI positively affect breastfeeding outcomes in the short, medium and long-term, but there is a dose-response relationship between the number of steps women are exposed to and breastfeeding outcomes [16]. There are more than 20,000 hospitals in 160 countries worldwide with BFI designated facilities [15, 17]. Despite existing evidence to support implementation of BFI guidelines, in Canada, there are only 23 hospitals or birthing centres, and 42 community health services that have achieved BFI designation [18]. To date, no facilities in Nova Scotia have achieved designation [18].

### Purpose

The primary goal of this study was to understand the process of evidence-informed decision-making within a regional community-based interorganizational network. The research questions aimed to describe the multiple perspectives towards the goal of achieving BFI designation and making breastfeeding the social norm. The mixed methods are nested within a case study research design.

### Research questions

1. What are the individual and external factors that influence the use of EIDM and the achievement of the network’s goals?
2. What are the interorganizational factors, including network structure, the sharing of information, and quality of relationships, that influence the network’s capacity for EIDM and its ability to achieve its goals?

## Methods

### Design

This research was guided by the tenets of case study research with embedded mixed methods for data collection and data analysis. Case study and mixed methods designs are often used in social and health sciences because they facilitate the investigation of complex social phenomena and a deeper understanding of context [19], especially with respect to group behavior and organizational processes [20]. The study methodology was adapted from a Canadian Institutes for Health Research-funded project examining EIDM and intersectoral public health networks [6]. Quantitative data were collected using a social networking questionnaire, and qualitative data were collected during the focus group discussion. The study obtained ethics approval from the local district health authority Research Ethics and Review Committee and the University of Ottawa Research Ethics Board (reference number: H09–11-07). Standard procedures were followed to ensure confidentiality, such as gaining informed consent.

### Setting

#### The case

The selected case was a rural network in Nova Scotia, focused on a collective goal of achieving BFI designation. The case was selected based on its relevance to the study questions while considering feasibility issues related to the time constraints of a 1 year research project. At the time of data collection, the Canadian province of Nova Scotia was comprised of 9 district health authorities, all of which were governed by the provincial Department of Health and Wellness, and ultimately reported to the provincial Minister of Health and Wellness. Each district had specific senior leadership teams and organizational structures that developed their own strategic plans and policies based on the needs of that district.

### Sample

The case was bound by time (2006–2011), geography (legislated boundaries of district health authority) and membership (guided by the network terms of reference). Data sources were people (network members- individuals representing various community organizations or district hospital units and public health departments) and documents related to the network’s activities (terms of reference and 5-year work plan). Recruitment was by email invitation to all members with a letter of information explaining the purpose of the study, the commitment required to participate, and a consent form that would be signed on the day of data collection.

### Measurement

The questionnaire reflects the roster method of whole network research where every network member was listed alphabetically by the authors prior to the completion of the questionnaire, rather than asking the network members to remember the names of members. Participants were then asked if they routinely interacted with the other members, even if they were absent on the day of data collection [21]. Questionnaire development regarding research and knowledge needs was guided by Haythornthwaite’s questionnaire [22] and was pilot tested and adapted by a national research collaborative [6]. Further, the questionnaire focused on asking participants about their relationship with member organizations in terms of trust, joint activities with network members, communication with network members, and common goals with respect to the focus of the work of the network. Interorganizational relationship questions related to collaborative practices were adapted from the work of Provan, Sebastian, and Milward [23]. Trust was measured indirectly through the members’ self-reporting of the quality of relationships with other members. Therefore, it was assumed that if members reported a high-quality relationship, they had a high level of trust in that individual [24].

### Data collection

Data collection occurred at a location determined by the co-chairs of the network. Eight participants from the network were present for data collection, which occurred over the span of several hours in 1 day. Participants were first asked to review the letter of information and informed consent document prior to data collection. Participants then completed the self-reported questionnaire. Following a short break, the focus group discussion began, which was audio recorded and facilitated by the first author using a focus group guide that was tested by the national research team previously mentioned [6], and adapted to match the purpose of this research and the context of this particular network. Following the completion of data collection, absent members of the network were invited to participate in individual interviews and complete the questionnaire that would be forwarded by email. There was no response from either member, and thus no further questionnaires were completed and only one focus group discussion occurred.

### Data analysis

**Fig. 1:**
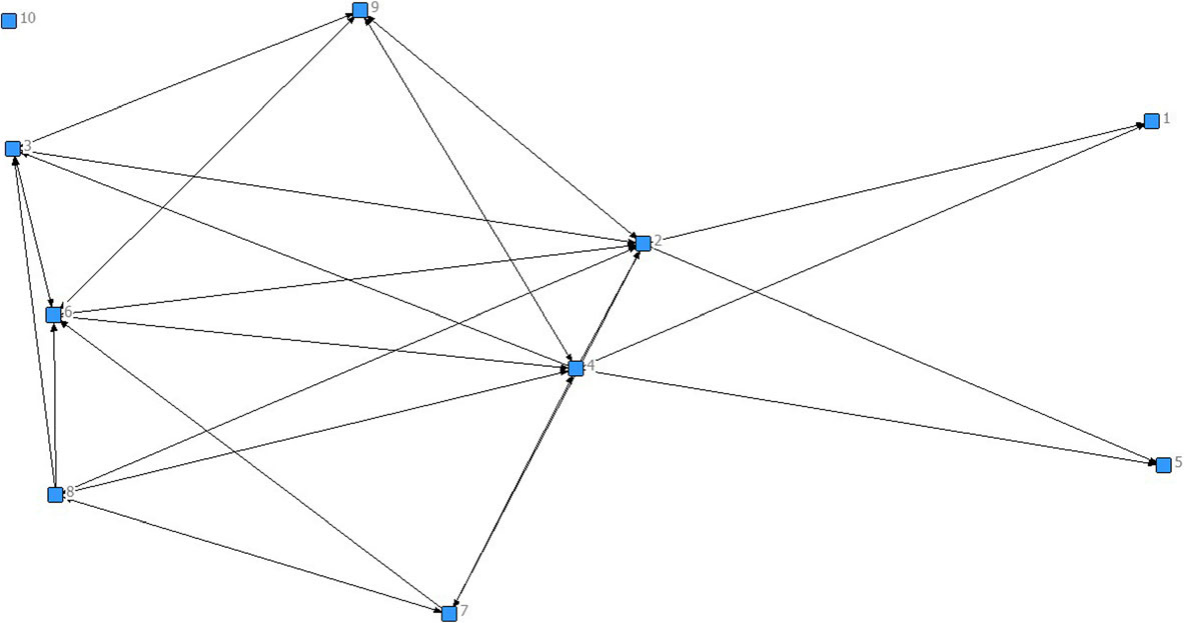
Common Goals. Network members were asked to identify other network members who “have common goals for the direction of the BFI committee”. Note: Each square (node) represents a network member, and the direction of the arrows indicates which member they identified as having common goals. The distance between nodes does not signify a stronger relationship. The position of each node is also not representative of the strength of the relationship. A reminder that 1 and 10 were not present for data collection, so no arrows will point away from them, but arrows will point towards them

**Fig. 2:**
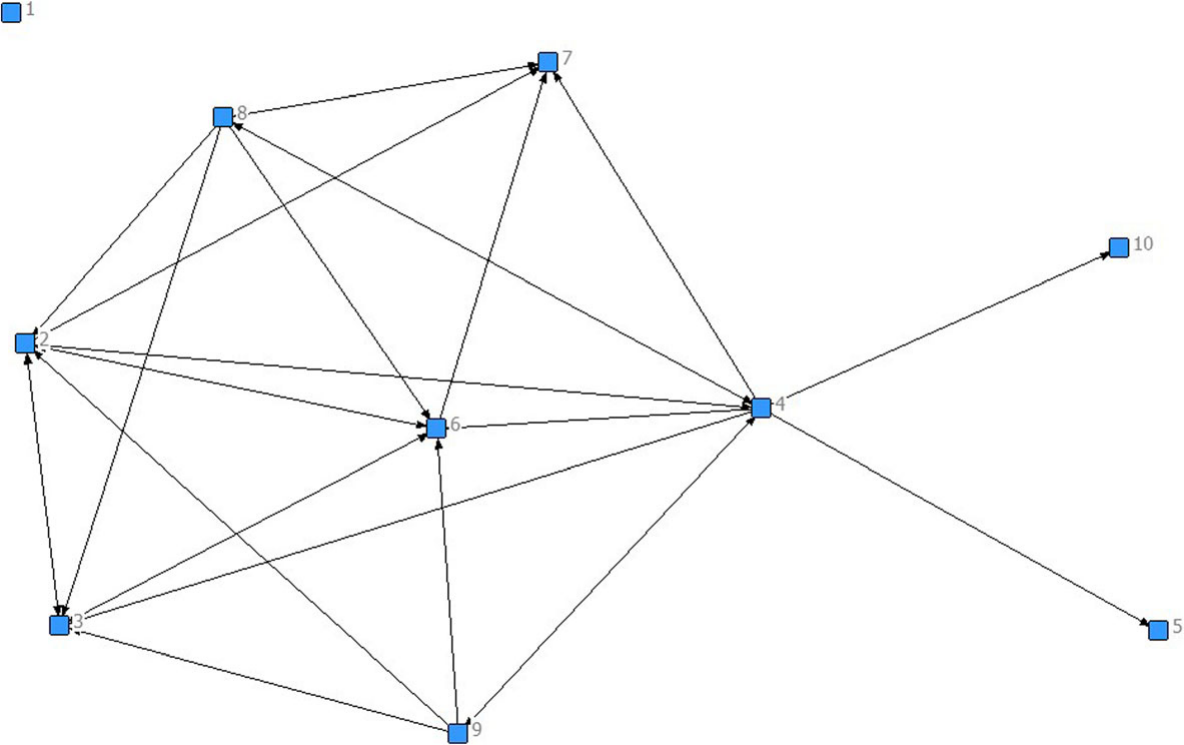
Providing Scientific Research Related to BFI. Network members were asked to identify other network members who “provide scientific research related to BFI”. Note: Each square (node) represents a network member, and the direction of the arrows indicates which member they identified as providing scientific research. The distance between nodes does not signify a stronger relationship. The position of each node is also not representative of the strength of the relationship. A reminder that 1 and 10 were not present for data collection, so no arrows will point away from them, but arrows will point towards them

Social Network Analysis (SNA) was used to analyze the questionnaire data. Responses to the SNA questionnaire were analyzed such that network-level measures were generated. Data were converted to matrices for analysis in UCINET 6©, a network analysis software package. The usual network metrics [25, 26] related to network structure (i.e., describing the distribution of relations among primary organizations) were computed for all interaction variables, based on the fact that organizations have multiple relationships with each other. NetDraw, a feature in UCINET 6©, was used to generate diagrams of the network. Typically, networks are represented graphically as a set of actors, or in this case, network members, who are connected by lines and arrows that highlight relationships between members. Each node, or square with an associated number represent the network member that in turn is on the network as a representative of one of the 5 hospital or community based organizations (see Figs. 1 and 2). For each question that was asked in the questionnaire, a graph was created to provide a visual representation of the answers from each network member. For example, for Fig. 1, the question was asked: Identify which network members have common goals (with you) for the direction of the BFI committee. What the graph shows, using arrows, is who each network has identified as having common goals. Members can name as many network members as are relevant to the question. Nodes that have no arrows leaving them are the two members who were not present for data collection. Other members were asked to still consider them when answering their questions, so you will see arrows pointing toward them, but there will never be arrows leaving these nodes.

Framework analysis was used to analyze the focus group data [27]. The focus group guide was adapted from the aforementioned study [6] to reflect the context of a rural health network. As per the concurrent triangulation approach in mixed methods research, quantitative and qualitative data were analyzed concurrently in an iterative and interactive manner, and equal weight was given to both forms of data when presenting results [19].

Scientific rigour was maintained through the use of several key strategies to ensure the trustworthiness of data, including: triangulation of data, peer debriefing, member checking, review of the raw transcript without identifiers by the research team, and using a data collection tool (questionnaire) that was adapted and pilot tested prior to this study [28].

## Results

### Sociodemographics of participants

Within this regional network, representatives from five of seven organizations participated. These eight members from five organizations participated in both the focus group and the completion of the survey. The two members who did not attend had conflicting schedules for the day of data collection, and did not respond to an invitation to do in-person interviews at a later date. All participants (*n* = 8) were female, and most were within the age range of 30–49 years. All participants had pursued some form of post-secondary education. Half of the participants in this study were Registered Nurses. Most participants (*n* = 6) were healthcare professionals. The most represented organization was the district health authority (*n* = 5), with three from Public Health Services, and two representing acute care maternal child services. The remaining participants represented community-based organizations and a university. A majority of the participants had been working in their current role for 10 years or less. Five participants had been members of the network for 6 months to 2 years. Most participants (*n* = 6) were in paid positions that supported their role as a network member while the remaining (*n* = 2) were network members emanating from the volunteer sector. Five of eight participants reported frequently or “almost always” participating in activities of this network.

The following paragraphs present the results of the analysis and interpretation of documents, focus group data, and SNA questionnaire. The role of the individual was difficult to capture due to the broader barriers that were considered outside of the network’s control being faced at the time of data collection. The key barriers and facilitators to EIDM and the achievement of the network goals were in fact at the external or system level, such as human and financial resources available to the network. In particular, leadership emerged as an overarching theme within the context of supporting EIDM in a network and assisting that network in achieving their goals. Within this overarching theme, resources and managerial presence were identified as issues that require the attention of multiple levels of leadership.

### Interorganizational factors

Key interorganizational factors, as assessed using the SNA questionnaire, included trust in relationships among network members and the link between trust and sharing information. When participants were asked about potential ways to strengthen relationships and build trust within the network, the following key factors were identified: effective communication, face-to-face meetings, pursuing a common goal, joint activities and subgroups, and informal discussions that occur outside of the meeting agenda. Upon review of the terms of reference from 2006 to 2011, it was noted that there had been fluctuation of membership, especially from Public Health. Relationships and trust require time to build, and the fluctuation of membership may be a barrier to sharing evidence. The following quote highlights the role that the structure of the network has on sharing evidence:

*And that’s what’s good about multidisciplinary. You know what other people do, you know they’re the expert on that, I can you know, use you as a resource and she’s got access to those, all those data, all that stuff, I know she’s got access to that where if she had something, she knows we’ve got our resources (Participant 6).*

Despite the stated commitment to a common goal during the focus group discussion, there were tensions among members about ways to achieve this goal. The tensions were apparent in the SNA results when participants were asked to identify members with whom they interacted regularly, shared information, and collaborated. The following quotation shows this tension as one participant questions the focus on BFI:

*But this committee as a whole, at the moment our workplan is entirely based around the ten steps … those are very much organizational, institution based, it isn’t the community piece. I know what the tenth step says, but it tends to be an afterthought in the process. We’re doing top down when we’re working on BFI, not grass roots up (Participant 3).*

Several participants reported the relevance of the goals of the network, to achieve BFI designation and normalize breastfeeding, in relation to unifying members and moving their agenda forward, for example: “*… everyone is marching on the same path. I find everyone wants the same outcome, no matter what level you work at. Having that clear vision of where we want it to go has been really helpful (Participant 7)”.* Figure 1 presents the SNA graph that depicts those who “have common goals for the BFI committee.” The graph clearly indicates, by simply noting that not all arrows are reciprocal, that the network does not completely agree on one vision for the network’s future. It is important to note that network members labeled “1” and “10” were not present at the time of data collection, so will never have arrows directed at other members. They may, however, have arrows pointing towards their node.

The impact of conflict between one member and the remaining network members was further revealed in the SNA questionnaire results. The member that is concerned about the lack of consideration of supports available in the community, was also reported by other members as not having common goals for the direction of the network. This network member also had no shared activities or programs with other members, and had a low level of trust by other members to keep their primary organization’s (the organization that each network member was representing- for example the the local maternal child unit, public health, the university, etc.) interests in mind during network activities and the pursuit of network goals. No members reported going to this organization for advice regarding BFI matters or receiving science-related research from them as it relates to breastfeeding in general or BFI.

Throughout the analysis process, there was a clear distinction between relationships among network members representing the local health authority versus those representing community-based organizations. Those individuals who were employed by the health authority seemed to form a “core group” of participants who showed a high level of mutual trust and shared joint activities and programs; they were relied upon to provide scientific research, to provide BFI-related advice, and to be resources when trouble-shooting about a new idea or when problem solving. The one exception to this was one community-based member who had a long-standing network membership. The remaining community-based organizations had little interaction with other organizations.

In addition to the WHO and UNICEF’s BFI guidelines [15], the following types of evidence were identified as supporting the workplan and other network activities: local statistics and reports, material from an 18-h breastfeeding course, documents and publications related to individuals’ professional organizations, provincial handouts and materials for clients, and the Health Canada website. The SNA graphs provided important information on the sharing of evidence within the network. Most members identified this same core group when asked from whom they would seek advice related to BFI matters, and who in the network would provide scientific research related to BFI (See Fig. 2 for the map that reveals who network members go to when searching for scientific research related to advice). This core group of members that were relied upon for evidence and advice were also the same members who were trusted to have a common goal with most network members.

### External factors

The majority of the identified barriers to EIDM and network goal achievement were related to external or system level factors, including human and financial resources, and in-person or network membership support from management. The one identified barrier to making breastfeeding the social norm in the district health authority was the time required for change to occur within the broader community. Human and financial resources were identified as the key barriers to implementing the programs and social marketing campaigns necessary to achieve the network’s goal. The following passage illustrates this barrier: “*… if you’re saying to me, we’ll hire somebody for a year, BFI would happen, because you would have somebody dedicated to do just that (Participant 2) …*” *.* Considerable discussion took place regarding the need for more tangible financial and human resource support for the BFI program, as identified by this participant *“You can’t have a strategic direction if you have a bunch of people trying to do it off the side of their desk (Participant 7)”.*

Two factors were reported as affecting individuals’ ability to participate in the EIDM process as well as contribute to the achievement of the network’s goals: competing priorities in the workplace and lack of time assigned to network activities. Participants reported time as the greatest barrier to sharing and putting evidence into practice. Throughout the focus group, participants frequently discussed recommendations for practice and interventions necessary to achieve the network’s goal and facilitate EIDM. All members agreed that a full-time BFI coordinator is required to put the workplan into action, as demonstrated in the following comment:

*I think again resources, looking at somebody that’s going to be a champion, that’s going to be able to have time to really look at a community, what we have with our tobacco coordinators, somebody that’s going to spread the word (Participant 2).*

The role of the coordinator was compared to a recent success with the provincial tobacco strategy [29]. This provincial initiative had a social marketing campaign and a provincial committee, while also having regional coordinators to implement, monitor, and evaluate programs. Participants were clear that they believed the success of the tobacco strategy was in large part due to the human and financial resources that were allocated, but also to the dedication to multiple levels of programming, from the individual to managers in health services. It was suggested that in order to mobilize these resources it was essential to have a manager as a member on the BFI network, as well as stronger general commitment to breastfeeding from the provincial government. The following quote illustrates the comparison between provincial support for the tobacco strategy and BFI:

*… so when we look at BFI designation, that’s how we see it as a BFI designator person you know, all across the province, do it together as a province, its like way more bang for your buck, and that’s what you do and so they did that for (tobacco strategy), and they had huge success with that and we have a lot to learn from them … (Participant 6).*

The results of this study suggest that members within a network who have had the opportunity to build trust are more likely to share knowledge and evidence. The act of sharing knowledge in itself is an activity that can build trust, thus contributing to a reciprocal relationship between trust and EIDM. Again, the key facilitators of trust were: organizations having a common goal, in-person meetings, and joint activities or programs.

### Rural challenges

When asked about adapting evidence to the local district, network members identified the challenge of making recommendations for practice that were typically developed in an urban area work in a rural setting. The following statement exemplifies their concerns:

*We’re in a rural setting and that’s a big component of it, some of the research or the articles or the projects are being rolled out in much larger centres, and so we have to think about how we roll that out here, and because we are rural it often does mean sometimes that we have less resources, technically we have less people out in the population, but we still have to do the same kind of work, and so that can be challenging, but I think sometimes its just trying to gear any of our programs or initiatives to a rural setting, and I don’t think people get how rural we are.*

Concerns regarding the challenges of living in a rural setting arose again when discussing the challenges of adapting the BFI guidelines to the district, as demonstrated by the following participant:

*I think of the mom, of a mom in xxx (rural community, population 806) (Statistics Canada, 2011) who has no other family members that have children, she is really the one that will not seek that support network. I really feel that it’s demographics with a lot of the people, you’ll see more issues in the rural (communities).*

Network members were asked what future research is needed to move forward with BFI. Their recommendations included a response to the rural challenge by identifying the barriers to breastfeeding duration specific to this district. Network members agreed that the district  has a diverse and unique population that requires specific interventions that are tailored to their needs.

As previously mentioned, the network maps of this study showed a preference for a core group of members, as noted by the increased number of reciprocal ties in the maps, and thus trust for these members. Following the member check, and after viewing the network maps, members verbalized an understanding of the way that relationships enhanced the success of the network. These results show potential for network maps to identify existing relationships and to be potentially used as a knowledge mobilization tool to both strengthen relationships and increase the likelihood of achieving network goals.

## Discussion

**Fig. 3:**
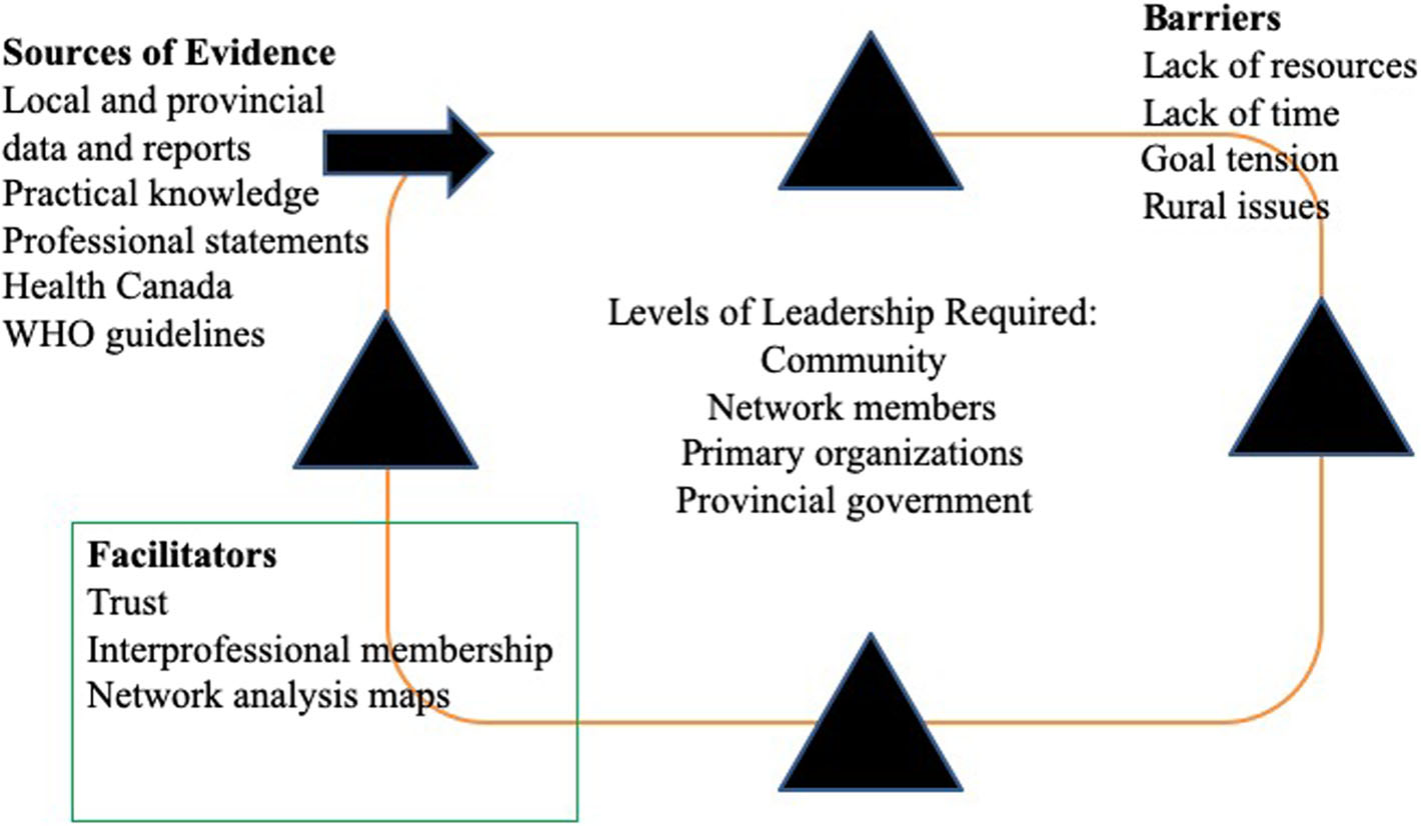
Facilitating EIDM and Goal Achievement in a BFI network

A conceptual framework (see Fig. 3) was developed to highlight the current study findings and existing empirical and theoretical evidence. The overarching theme of leadership and, in particular, the need for multiple levels of leadership, is shown at the center of the model, which is shaped as a network. The multiple levels of leadership identified in this study are: community, network members, primary organizations, and the provincial government. The key barriers and facilitators to EIDM and achieving BFI designation are identified within the network continuum. The sources of evidence used by the network to make decisions are included in the framework.

The study findings indicated that leadership was a crucial factor in supporting EIDM and network goals. An evaluation of the implementation of the provincial breastfeeding policy in Nova Scotia provides important support for these findings [30]. The provincial policy was launched in 2005 with three objectives: “provide leadership for protection, promotion, and support of breastfeeding; improve the health status of mothers and babies by increasing initiation and duration rates; and support the implementation of BFI [31]”. The policy was meant to leverage support at provincial and district levels, and the provincial steering committee was accountable for its implementation [30]. An evaluation of the implementation of the policy revealed that the current efforts are insufficient in Nova Scotia’s “unsupportive breastfeeding culture[30]”. Further, an integrative review of the barriers, facilitators and recommendations related to implementing BFI revealed similar results to this study. The results identified the following priority issues that need to be addressed when pursuing BFI designation: endorsement of administrators and policy makers, leadership to support the change process, health care worker training, marketing influence of the formula company, and integrating hospital and community health services [32]. Similarly, Alakaam and colleagues reported that the main barriers to implementation of the BFI were resistance to new policies, limited financial and human resources, and lack of support from national and state governments [33].

The current study highlighted the relational aspect of sharing knowledge as a key facilitator in the implementation of the BFI guidelines. Many types of evidence were used by the BFI network in its decision-making. The benefits of interorganizational collaboration in relation to EIDM were identified in a case study of a cancer research network in the United States [34]. As was reported by participants in this case study, collaborations within a network (especially joint activities on other committees and projects) improved knowledge exchange [34, 35].

With respect to network structure and membership, fluctuation of representation from organizations may be a barrier to developing trusting relationships [36]. The rationale for requesting the presence of a senior manager may have been a reaction to fluctuating membership and reflective of the need for decision-making ability and some consistency. The request for management membership and engagement in the network is supported by the literature, especially in early network formation [37]. There is a need for clearer direction regarding the role of management and decision-makers in goal-directed health networks.

According to stakeholders, the following key factors are influential in improving the success of clinical networks: building relationships, effective leadership, use of strategic evidence-based work plans, adequate resources, and the ability to implement and evaluate network initiatives [38]. These factors are consistent with the facilitators identified in this study. The last strategy, related to the implementation and evaluation of initiatives, was discussed in the context of needing resources and managerial support in order to achieve the network goal of BFI designation. Experts recommend that leadership be engaged at multiple levels, as shown in the proposed conceptual framework (see Fig. 3), to assist the network in achieving its goal [38]. Hiring a BFI coordinator and having a senior manager capable of making human resource and budget decisions as a member of the network will not alone address the network level barriers that are present in the flow of information, the tensions in relationships regarding the goals and direction of the network, and the more systemic lack of commitment from the senior leadership team that has the power to allocate sustainable funding to the network.

Consistent with this current study, Varda and Retrum [39] used SNA to identify the structural and organizational characteristics of public health collaboratives with 11 networks in the United States. External and interorganizational dynamics influenced the outcomes of the networks [39]. Increasing reciprocal relationships within the network, as shown among the most trusted network members, would encourage a greater flow of information [39]. The participation of network members in meetings and in leading subcommittees also increases the flow of information. With respect to the regional network, others have found that paid staff and members with specific health expertise provided by an organization were more trusted than unpaid or volunteer members [39]. Similar patterns were seen in the network maps of this current study as noted by the increased number of reciprocal ties, and thus trust, demonstrated among predominantly district health authority staff.

The findings of the present study have potential implications for enhancing the use of EIDM as other BFI networks work toward designation. The results suggest that in the early phases of the BFI process, the network may require multi-level leadership, the opportunity for relationship building and the sharing of various forms of evidence. Given the perceived importance of the key role of network leadership, especially managerial presence described by the participants in this study, future guideline implementation models could articulate more clearly the role of different types of leaders. There is little direction from the WHO or Breastfeeding Committee for Canada regarding which type of collaboration is effective, and which resources are necessary for achieving BFI.

It is important to note that this study highlighted barriers to breastfeeding promotion that are unique to rural communities, and these findings contribute to a limited number of studies that examine this aspect of working towards BFI. Smith [40] recognizes that breastfeeding is a complex issue that is deeply connected to social inequities and that a social justice approach to breastfeeding promotion is required in order to address intersecting inequities such as gender, race and poverty [40]. Further research is needed to understand the nuances of adapting national guidelines in rural settings, in addition to the unique barriers to providing breastfeeding support and promotion.

### Limitations

Limitations of this study include the small sample size and the self-reporting nature of focus groups and the survey, which may have resulted in survey responses with a social desirability bias. The focus group occurred at one point in time, limiting the knowledge of how this emerging network evolved over time, although a document review of 5 years of achievements was helpful in outlining the process. Despite the small sample size of one network, the response rate was 80% of the whole network, providing a representative sample of this particular network. The researcher’s previous experience with the network and personal nursing practice experience provided important knowledge and context for adapting tools and analyzing the data [20]. This study contributes to a limited knowledge base on the challenges of implementing BFI in rural areas and in conducting SNA analyses for knowledge translation.

## Conclusion

A greater understanding of the barriers and facilitators to EIDM in community-based networks especially in rural regions is lacking in the current literature. This study shows the potential for network maps to be used as a knowledge mobilization tool, and future studies should investigate their use over time in community networks.

## Abbreviations

BFI: Baby Friendly Initiative
EIDM: Evidence-Informed Decision-Making
SNA: Social Network Analysis
